# *Macrophage-Stimulating 1* Polymorphism rs3197999 in Pediatric Patients with Inflammatory Bowel Disease

**DOI:** 10.3390/medicina60081243

**Published:** 2024-07-31

**Authors:** Jan Brylak, Jan K. Nowak, Emilia Dybska, Aleksandra Glapa-Nowak, Jarosław Kierkuś, Marcin Osiecki, Aleksandra Banaszkiewicz, Andrzej Radzikowski, Anna Szaflarska-Popławska, Jarosław Kwiecień, Anna Buczyńska, Jarosław Walkowiak

**Affiliations:** 1 Department of Pediatric Gastroenterology and Metabolic Diseases, Poznan University of Medical Sciences, 60-572 Poznan, Polandjan.nowak@ump.edu.pl (J.K.N.); glapa@ump.edu.pl (A.G.-N.); 2 Department of Gastroenterology, Hepatology, Feeding Disorders and Pediatrics, The Children’s Memorial Health Institute, 04-730 Warsaw, Poland; 3 Department of Pediatric Gastroenterology and Nutrition, Medical University of Warsaw, 02-091 Warsaw, Poland; 4 Department of Pediatric Endoscopy and Gastrointestinal Function Testing, Collegium Medicum in Bydgoszcz, Nicolaus Copernicus University in Torun, 85-094 Bydgoszcz, Poland; 5 Department of Pediatrics, Faculty of Medical Sciences in Zabrze, Medical University of Silesia, 40-055 Katowice, Poland; 6 Department of Pediatrics, Faculty of Medical Sciences in Katowice, Medical University of Silesia, 40-055 Katowice, Poland

**Keywords:** Crohn’s disease, ulcerative colitis, pediatrics, steroids, *MST1*, height, polymorphism, infliximab

## Abstract

*Background and Objectives:* Inflammatory bowel disease (IBD), which includes Crohn’s disease (CD) and ulcerative colitis (UC), often necessitates long-term treatment and hospitalizations and also may require surgery. The *macrophage-stimulating 1* (*MST1)* rs3197999 polymorphism is strongly associated with the risk of IBD but its exact clinical correlates remain under investigation. We aimed to characterize the relationships between the *MST1* rs3197999 genotype and the clinical characteristics in children and adolescents with IBD within a multi-center cross-sectional study. *Materials and Methods:* Clinical data included serum C-reactive protein (CRP), albumin, activity indices (PUCAI, PCDAI), anthropometric data, pharmacotherapy details, surgery, and disease severity. Genotyping for rs3197999 was carried out using TaqMan hydrolysis probes. *Results:* The study included 367 pediatric patients, 197 with Crohn’s disease (CD) (40.6% female; a median age of 15.2 years [interquartile range 13.2–17.0]) and 170 with ulcerative colitis (UC) (45.8% female; a median age of 15.1 years [11.6–16.8]). No significant relationships were found between *MST1* genotypes and age upon first biologic use, time from diagnosis to biological therapy introduction, PUCAI, PCDAI, or hospitalizations for IBD flares. However, in IBD, the height Z-score at the worst flare was negatively associated with the CC genotype (*p* = 0.016; CC: −0.4 [−1.2–0.4], CT: −0.1 [−0.7–0.8], TT: 0.0 [−1.2–0.7)]). The TT genotype was associated with higher C-reactive protein upon diagnosis (*p* = 0.023; CC: 4.3 mg/dL [0.7–21.8], CT 5.3 mg/dL [1.3–17.9], TT 12.2 mg/dL [3.0–32.9]). *Conclusions:* This study identified links between *MST1* rs3197999 and the clinical characteristics of pediatric IBD: height Z-score and CRP. Further studies of the associations between genetics and the course of IBD are still warranted, with a focus on more extensive phenotyping.

## 1. Introduction

Inflammatory bowel disease (IBD), which includes Crohn’s disease (CD) and ulcerative colitis (UC), affects approximately 0.3% of the population of Poland [1] and up to 1% in the most highly developed countries [2]. The course of IBD varies individually [3], but a large share of patients require hospitalizations, with a trend for increased in-hospital management in many countries globally [4]. The disease affects both pediatric and adult populations, burdening the caregivers and the healthcare systems [5]. Its long-term treatment focuses on controlling the symptoms and does not always prevent the need for surgery. Moreover, pharmacotherapies have limited efficacy, exhibit side effects, and may lose their beneficial effects with time as in the case of anti-tumor necrosis factor alpha (TNF-alpha) biologics [6]. Furthermore, dietary treatment, whilst efficacious in pediatrics, remains relatively challenging in practice in adult patients with CD [7,8] and still requires further research in UC [9]. The disease not only results from lifestyle but can also impact it by limiting physical [10] and social activities. The most recent push to predict response to individual IBD treatments is not always bearing fruit [11], but efforts continue to enable precision medicine in IBD, with some promising results [12]. These findings build on IBD omics, including IBD genomics, where the rapid growth in knowledge [13] has not always been accompanied by independent investigations of relationships between individual single-nucleotide polymorphisms (SNPs) and characteristics of IBD.

The SNP (single-nucleotide polymorphism) rs3197999 (C/T; missense) is located in the last exon of *MST1* (encoding *macrophage-stimulating 1*; 487 aminoacids), and is less than 1 kbp following *acylaminoacyl-peptide hydrolase* (*APEH)*, encoded on the opposite strand [14]. It was found to be the top SNP in this genomic region following an IBD genetics meta-analysis [13], with a similar strength of relationships for both CD and UC. The overall statistical significance for the genetic relationship between rs3197999 and IBD was the 8th strongest of 241 loci, giving *MST1* the place among such key IBD genes as *IL23R*, *NOD2*, *CARD9*, and *IL10*. Of note, rs3197999 is also a risk factor for primary sclerosing cholangitis, which may accompany UC [15,16] and is associated with a gain of function of MST1 [17]. Yet, *MST1* also attracts attention across a broad spectrum of disciplines not related to the strongest findings from genome-wide association studies, including cardiology, diabetology, neurology, and oncology [18,19,20]. Especially in this last context, much stress is placed on the role of MST1 in the Hippo tumor suppressor pathway, which regulates cell proliferation [21].

There are, however, also reports of the role of MST1 in immunity, with reduced peripheral T cells described in *Mst1*-null mice, potentially due to early apoptosis, especially in the case of more easily proliferating naïve T cells, potentially providing a link to the Hippo pathway [22]. *Mst1*−/− mice may be susceptible to T cell-related autoimmunity because of insufficient antigen recognition during thymic selection [23], although data on this topic are conflicting [22]. Importantly, the development of FoxP3+ CD4+ Tregs was found to be impaired in *Mst1*-null mice through the insufficient post-translational acetylation of Foxp3 [24]. The influence of MST1 on immunity may involve known IBD actors such as LFA-1 and ICAM-1 and processes such as cytoskeleton dynamics [25].

Therefore, *MST1* is one of the most important genetic contributors to the development of IBD and its role is not entirely clear [26]. We aimed to provide new data on the relationship between *MST1* and the clinical picture of pediatric IBD.

## 2. Materials and Methods

The study is based on samples gathered in the years 2016–2019 at seven pediatric university hospitals in Poland, in the cities of Poznań (n = 1), Warsaw (n = 2), Wrocław (n = 1), Zabrze (n = 1), Katowice (n = 1), and Bydgoszcz (n = 1). The inclusion criteria were (i) IBD diagnosed according to current guidelines, (ii) age between 3 and 18 years, and (iii) the absence of life-threatening symptoms [27]. Informed consent was obtained from parents or legal guardians of all the children and adolescents and from adolescents aged 16 years or older. The study was carried out within the scope of formal approval by the Bioethical Committee at Poznan University of Medical Sciences (960/15, with amendments). The study respected the rules set out in the Declaration of Helsinki [28].

The basic collected data were age upon diagnosis and inclusion, gender, type of IBD (UC or CD, without IBD-unspecified), and a range of parameters upon diagnosis and at the worst flare: serum C-reactive protein (CRP); albumin; Pediatric Ulcerative Colitis Index (PUCAI) [29] or Pediatric Crohn’s Disease Activity Index (PCDAI) [30]; and Z-scores for body mass, height, and body mass index (BMI), based on local reference values. Severity was thought to be reflected by the employed treatment: systemic steroids, azathioprine, methotrexate, ciclosporin, biologics (infliximab, adalimumab), and also more specifically, the time from diagnosis to starting biological therapy, age at the introduction of therapy with a biologic, the need for any IBD-related surgery, the number of hospitalizations for IBD flares, and in the case of patients with a disease duration > 1 year, also the number of hospitalizations (and days of hospitalization) for IBD flares per year.

DNA was obtained from whole blood sampled in tubes with EDTA using a microcolumn-based kit (Blood Mini, A&A Biotechnology, Gdynia, Poland). Genotyping was based on the use of a TaqMan probe with the TaqMan Genotyping Master Mix (both from Thermo Fisher Scientific Inc., Waltham, MA, USA). The CFX96 thermocycler system (Bio-Rad, Hercules, CA, USA) was used to carry out quantitative PCR reactions and discriminate alleles based on plots. The four runs included negative control. The number of cycles was 41. Samples with fluorescence close to zero were excluded prior to analyses (7 samples). The plot of the sample fluorescence and genotyping is attached as Supplementary Figure S1.

Data were analyzed to explore if the homozygosity of the major or minor allele (dominant/recessive model) was associated with the phenotype. The analyses were carried out using the Mann–Whitney U test because of the encountered non-normal distributions. Additionally, the Kruskal–Wallis test was applied to compare variable values between genotypes. Two-way tables were analyzed using Fisher’s test. Two-sided *p* values are reported. The significance (alpha) threshold was set at 0.05. Statistical analyses were carried out using PQStat (PQStat, Poznan, Poland). The data were analyzed separately for CD, UC, and also in the entire IBD group to achieve the maximum power of analysis with regard to common variables.

The list of abbreviations can be found in the Supplementary Materials (Supplementary Table S1).

## 3. Results

Three hundred sixty-seven children and adolescents were included in the analysis (197 with CD; 170 with UC). The CD and UC groups were similar in age upon diagnosis (or inclusion), sex, and frequency of rs3197999 alleles. The median duration of the disease was above 2 years and the data concerning hospitalizations were available for more than half of the patients in both CD and UC. Over half of pediatric patients in both groups received azathioprine. However, the frequency of the use of anti-TNF biologics was lower in UC, which is discussed in limitations.

No relationships were found between *MST1* genotypes and age upon the first biological therapy or time from diagnosis to biological therapy, PUCAI, PCDAI, or hospitalizations for IBD flares (Table 1). In UC, a marginally lower height Z-score at the worst flare was found in the homozygous rs3197999 CC genotype relative to other genotypes (Table 2). In CD, the CC genotype was both positively related to the use of systemic steroids and more common in female patients (Table 3). In IBD overall, the height Z-score at the worst flare was negatively associated with the CC genotype but the TT genotype was correlated with higher C-reactive protein upon diagnosis (Table 4).

## 4. Discussion

This cross-sectional study investigated the relationship between *MST1* SNP rs3197999 and the clinical characteristics of IBD in pediatric patients across Poland. The need for study resulted from the high burden of disease and strong links between the aforementioned SNP and the risk of IBD, with compelling molecular evidence for the involvement of *MST1* in immunity. The identification of relationships between specific clinical characteristics and the polymorphism could help in the understanding of its role and the involved mechanisms (with potential implications for treatment) and might provide a new tool for risk assessment in the pediatric population.

The rs3197999-CC genotype was negatively associated with height Z-score at the worst flare in IBD, possibly reflecting a greater impact of disease on development, potentially confounded by the more frequent use of steroids in CD (which negatively impacts growth [31]). However, this result implicating greater severity in rs3197999-CC appeared discordant with higher CRP upon diagnosis in patients with IBD with the rs3197999-TT genotype. These observed differences, albeit relatively small, provide new insights into the role of *MST1* in IBD.

Previous studies provide strong evidence for the link between *MST1* and IBD. In a study of almost 35 000 patients with IBD, *MST1* rs3197999 was associated with the risk of UC and younger age upon its diagnosis, as well as with the ileal location of CD (vs. colonic, OR 1.10 [1.02–1.19] in [32] and vs. control OR 1.5 [1.1–2.1] in [33]). In yet another study, rs3197999 was associated with early-onset UC, distal colitis, and a reduced risk of extraintestinal manifestations [34]. In patients with CD, *MST1* rs3197999 conferred a higher risk of perianal disease (OR 1.19 [1.01–1.41) [35]. Importantly, *MST1* IBD risk loci were replicated in populations globally, including China [36], South Asia [37] and Eastern Europe [38].

Genomic analyses of three large cohorts suggested that higher MST1 levels may be protective against IBD [26]. The minor allele of rs3197999 correlated negatively with the serum concentration of MST1 [39], and the relationship between SNPs in the *MST1* locus and their relationship with IBD risk overlapped. The lower serum concentration may be explained by the lower stability of the mutant protein [40]. The SNP may have gain-of-function effects with regard to macrophage inflammatory activity [17].

The investigated SNP was also found to be a risk factor for extrahepatic cholangiocarcinoma, and this was independent of the diagnosis of primary sclerosing cholangitis [41]. This suggests a role for MST1 in the bile ducts, which is possibly related to inflammation that may underlie different types of disease. Additional insight into rs3197999 is provided by results implicating it as a modifying factor (relationship quantitative trait locus) of genetically predicted insulin concentration and sensitivity to insulin [42]. Analyses of *MST1* beyond rs3197999 link it also to other diseases, which is illustrated by *MST1* rs9858542—a risk factor for both CD (more frequent homozygosity of the minor allele) and ankylosing spondylitis (more frequent heterozygous genotype, compared with controls) [43].

*GPX1* (glutathione peroxidase 1) neighbors *MST1* (within 300 kbp), and some studies pointed towards its involvement in IBD, possibly through its antioxidant functions. Evidence comes from Mendelian randomization [44] and genetic analyses [45]. Notwithstanding the limitations related to potential linkage disequilibrium and difficulties in meeting assumptions of Mendelian randomization, in a specific *Gpx1*-null and *Gpx2*-null murine model (B6), the development of ileocolitis was facilitated [46]. Also, in patients with IBD, the activity of GPX1 decreased, but this may be an epiphenomenon [47] similar to the reduced *MST1* expression in the rectum in UC [48]. Although there is some evidence for the involvement of *GPX1* (or potentially also *BSN* and *APEH*), research supports the role of *MST1* most strongly.

In the earlier part of the study period, the treatment with anti-TNF agents was not easily accessible for pediatric UC in Poland, and, therefore, their use could discriminate the most severe cases of IBD. One of the limitations is the lack of a healthy control group to compare the prevalence of the SNP, and the lack of study of MST1 serum levels or *MST1* methylation. This study utilized data like height Z-scores, CRP levels, and the use of specific treatments, which might not capture the full clinical complexity of IBD. Moreover, it does not include fecal calprotectin level assessment, which was not systematically collected. The median disease duration was above 2 years, which might not be sufficient to observe long-term outcomes and the full impact of *MST1* genotypes on disease progression and treatment response. However, this study also provides interesting insights into such aspects as the age of IBD diagnosis in Poland and the most commonly used medications, including the high frequency of azathioprine use.

## 5. Conclusions

This study has found few links between *MST1* rs3197999 and clinical characteristics of IBD in pediatric patients. The *MST1* rs3197999-CC genotype was associated with a reduced height Z-score upon the worst flare and in CD, it could be related to the more frequent use of steroids. A link was also identified between this polymorphism and CRP upon diagnosis. However, no relationships were found between rs3197999 and PUCAI, PCDAI, hospitalizations, and the time from diagnosis to biological treatment. Further studies of the links between genetics and the course of IBD are still warranted, with a focus on more extensive phenotyping. They are likely to yield insights useful for understanding disease complexity and personalizing clinical care.

## Figures and Tables

**Table 1:** Group characteristics. Q1–Q3: quartiles 1–3.

	Ulcerative Colitis			Crohn’s Disease		
Analyzed Variables	n	%	Median (Q1–Q3)	n	%	Median (Q1–Q3)
*MST1* rs3197999 major homozygous CC	170	51.7%		197	52.8%	
*MST1* rs3197999 heterozygous CT	170	38.3%		197	33.5%	
*MST1* rs3197999 minor homozygous TT	170	10.0%		197	13.7%	
Age at diagnosis [years]	169		11.9 (7.8–14.8)	196		12.2 (9.9–14.2)
Age at inclusion [years]	162		15.1 (11.6–16.8)	196		15.2 (13.2–17.0)
Duration of the disease [years]	157		2.2 (0.6–3.9)	194		2.3 (1.0–4.5)
Female	170	45.8%		197	40.6%	
CRP at diagnosis [mg/L]	157		2.3 (0.5–9.6)	192		12.1 (2.0–28.7)
CRP at worst flare [mg/L]	140		2.7 (0.8–11.0)	172		13.5 (3.0–31.9)
Albumin at diagnosis [g/dL]	139		4.1 (3.7–4.4)	171		3.8 (3.5–4.3)
Albumin at worst flare [g/dL]	130		4.2 (3.8–4.4)	166		3.9 (3.5–4.2)
Mass Z-score at diagnosis	158		−0.5 (−1.1–0.2)	187		−0.7 (−1.4–0.0)
Height Z-score at diagnosis	154		0.0 (−0.7–0.7)	187		−0.3 (−1.3–0.5)
BMI Z-score at diagnosis	154		−0.4 (−1.0–0.2)	187		−0.7 (−1.4–0.0)
Mass Z-score at worst flare	139		−0.5 (−1.0– 0.4)	166		−0.8 (−1.4– −0.1)
Height Z-score at worst flare	139		0.0 (−0.7–0.8)	167		−0.3 (−1.3–0.3)
BMI Z-score at worst flare	138		−0.6 (−1.1–0.3)	166		−0.8 (−1.4–0.2)
PCDAI at diagnosis				176		30.0 (22.5–47.5)
PUCAI at diagnosis	147		35.0 (30.0–60.0)			
PCDAI at worst flare				160		40.0 (30.0–52.5)
PUCAI at worst flare	135		55.0 (40.0–65.0)			
Systemic steroids	170	74.1%		197	54.8%	
Azathioprine	169	58.5%		197	79.7%	
Methotrexate	169	3.5%		197	8.6%	
Ciclosporin	169	11.2%		197	3.0%	
Biologics	170	27.0%		197	50.7%	
Infliximab	167	25.1%		196	48.9%	
Adalimumab	167	5.9%		196	8.1%	
Months from diagnosis to biological therapy	44		13.5 (8.8–26.9)	96		14.1 (6.0–29.2)
Age at first biological therapy	45		12.1 (7.4–15.3)	96		13.6 (11.8–15.1)
IBD-related surgery	170	2.3%		197	13.2%	
Time hospitalized for IBD flare	148		2.0 (1.0–3.0)	172		1.0 (1.0–2.0)
Hospitalizations for IBD flare per year	94		0.7 (0.3–1.3)	130		0.5 (0.2–0.8)
Days hospitalized for IBD flare per year	94		4.7 (1.8–8.9)	129		3.8 (1.3–7.2)

BMI—body mass index; CC—cytosine/cytosine, CRP—C-reactive protein; CT—cytosine/thymine, IBD—inflammatory bowel disease; PCDAI—Pediatric Crohn’s Disease Activity Index; PUCAI—Pediatric Ulcerative Colitis Activity Index; TT—thymine/thymine, Z-score—number of standard deviations that the result differs by from the mean of the population at given age.

**Table 2:** Characteristics of children and adolescents with ulcerative colitis dependent on *MST1* rs3197999 genotype. The Mann–Whitney U test, the Kruskal–Wallis test (K-W: comparison of three groups), and Fisher’s test were used for comparisons. Q1–Q3: quartiles 1–3. Statistically significant results are marked with asterisk (*).

				CC			CT			TT		
Analyzed Variables	CC vs. Other *p*	TT vs. Other *p*	K-W *p*	n	%	Median (Q1–Q3)	n	%	Median (Q1–Q3)	n	%	Median (Q1–Q3)
Age at diagnosis [years]	0.189	0.162	0.058	88		11.6 (7.1–14.5)	64		13.2 (9.7–15.0)	17		9.2 (5.5–13.6)
Age at inclusion [years]	0.084	0.488	0.224	86		14.2 (11.8–16.3)	61		15.9 (11.6–17.1)	15		15.6 (11.5–17.6)
Duration of the disease [years]	0.155	0.278	0.089	82		2.3 (0.8–4.9)	60		1.8 (0.3–3.2)	15		2.3 (1.7–5.3)
Female	0.355	0.799		88	42.0%		65	52.3%		17	41.2%	
CRP at diagnosis [mg/L]	0.080	0.619	0.215	81		1.8 (0.4–8.2)	62		3.4 (1.2–9.1)	14		4.8 (0.6–28.7)
CRP at worst flare [mg/L]	0.515	0.434	0.476	69		2.7 (1.0–6.4)	60		2.8 (0.8–16.9)	11		1.1 (0.6–10.1)
Albumin at diagnosis [g/dL]	0.694	0.557	0.817	69		4.1 (3.6–4.5)	57		4.1 (3.7–4.4)	13		3.9 (3.7–4.2)
Albumin at worst flare [g/dL]	0.854	0.519	0.806	60		4.2 (3.9–4.4)	58		4.2 (3.8–4.4)	12		4.3 (3.8–4.7)
Mass Z-score at diagnosis	0.197	0.585	0.430	79		−0.6 (−1.2–0.2)	63		−0.4 (−0.9–0.1)	16		−0.2 (−0.9–0.2)
Height Z-score at diagnosis	0.035 *	0.256	0.099	76		−0.2 (−1.0–0.5)	62		0.2 (−0.4–0.9)	16		0.4 (−0.4–1.0)
BMI Z-score at diagnosis	0.643	0.379	0.633	76		−0.5 (−1.1–0.2)	62		−0.5 (−0.9–0.1)	16		−0.3 (−0.9–0.6)
Mass Z-score at worst flare	0.262	0.726	0.530	67		−0.6 (−1.1–0.1)	61		−0.5 (−0.9–0.7)	11		−0.8 (−0.9–0.7)
Height Z-score at worst flare	0.021 *	0.222	0.058	67		−0.4 (−1.0–0.4)	61		0.0 (−0.4–0.9)	11		0.2 (0.0–0.9)
BMI Z-score at worst flare	0.937	0.993	0.996	66		−0.7 (−1.0–0.1)	61		−0.5 (−1.2–0.6)	11		−0.4 (−1.1–0.5)
PUCAI at diagnosis	0.901	0.214	0.394	73		45.0 (30.0–65.0)	61		45.0 (30.0–65.0)	13		55.0 (40.0–60.0)
PUCAI at worst flare	0.734	0.374	0.545	63		50.0 (40.0–67.5)	60		52.5 (39.4–65.0)	12		62.5 (42.5–66.3)
Systemic steroids	0.295	0.564		88	70.5%		65	76.9%		17	82.4%	
Azathioprine	1	0.795		88	59.1%		64	56.3%		17	64.7%	
Methotrexate	1	0.476		88	3.4%		64	3.1%		17	5.9%	
Ciclosporin	0.339	1		88	13.6%		64	7.8%		17	11.8%	
Biologics	0.605	0.402		88	25.0%		65	27.7%		17	35.3%	
Infliximab	1	0.375		88	25.0%		62	22.6%		17	35.3%	
Adalimumab	0.750	1		88	6.8%		62	4.8%		17	5.9%	
Months from diagnosis to biological therapy	0.834	0.205	0.411	21		13.0 (9.1–26.9)	17		15.8 (11.1–27.0)	6		7.4 (3.8–21.2)
Age at first biological therapy	0.348	0.442	0.563	21		13.1 (9.9–15.4)	18		11.3 (6.9–14.8)	6		8.5 (4.6–14.5)
IBD-related surgery	0.353	0.050		88	1.1%		65	1.5%		17	11.8%	
Times hospitalized for IBD flare	0.818	0.886	0.970	74		2.0 (1.0–3.8)	62		2.0 (1.0–3.0)	12		2.0 (1.0–2.3)
Hospitalizations for IBD flare per year	0.064	0.685	0.175	48		0.6 (0.2–1.0)	37		0.8 (0.5–1.6)	9		0.9 (0.3–1.7)
Days hospitalized for IBD flare per year	0.148	0.454	0.334	48		4.6 (1.2–7.7)	37		6.1 (2.5–9.7)	9		6.9 (3.1–14.0)

BMI—body mass index; CRP—C-reactive protein; IBD—inflammatory bowel disease; PUCAI—Pediatric Ulcerative Colitis Activity Index.

**Table 3:** Characteristics of children and adolescents with Crohn’s disease dependent on *MST1* rs3197999 genotype. The Mann–Whitney U test, the Kruskal–Wallis test (K-W: comparison of three groups), and Fisher’s test were used for comparisons. Q1–Q3: quartiles 1–3. Statistically significant results are marked with an asterisk (*).

				CC			CT			TT		
Analyzed Variables	CC vs. Other *p*	TT vs. Other *p*	K-W *p*	n	%	Median (Q1–Q3)	n	%	Median (Q1–Q3)	n	%	Median (Q1–Q3)
Age at diagnosis [years]	0.550	0.808	0.835	103		12.1 (9.5–14.6)	66		12.2 (9.4–14.1)	27		12.4 (10.8–13.3)
Age at inclusion [years]	0.749	0.892	0.907	103		15.2 (13.4–17.1)	66		15.2 (12.8–16.8)	27		15.0 (13.4–17.4)
Duration of the disease [years]	0.721	0.316	0.600	102		2.1 (0.9–4.5)	65		2.3 (1.0–4.6)	27		3.4 (1.5–4.4)
Female	0.029 *	0.291		104	48.1%		66	33.3%		27	29.6%	
CRP at diagnosis [mg/L]	0.872	0.102	0.222	101		12.0 (1.9–29.6)	65		9.4 (1.5–19.8)	26		17.1 (6.8–30.6)
CRP at worst flare [mg/L]	0.797	0.300	0.569	92		14.1 (2.8–31.9)	56		11.3 (2.0–30.1)	24		17.4 (5.4–32.9)
Albumin at diagnosis [g/dL]	0.326	0.882	0.591	89		3.8 (3.4–4.3)	58		3.9 (3.7–4.3)	24		4.0 (3.7–4.1)
Albumin at worst flare [g/dL]	0.737	0.594	0.706	88		3.9 (3.6–4.3)	54		3.9 (3.4–4.1)	24		4.0 (3.6–4.1)
Mass Z-score at diagnosis	0.904	0.117	0.199	99		−0.8 (−1.4–−0.1)	62		−0.7 (−1.2–0.2)	26		−1.1 (−1.5– −0.6)
Height Z-score at diagnosis	0.285	0.307	0.428	99		−0.4 (−1.2–0.4)	62		−0.1 (−1.3–0.6)	26		−0.7 (−1.4–0.6)
BMI Z-score at diagnosis	0.794	0.251	0.364	99		−0.7 (−1.4–0.0)	62		−0.7 (−1.2–0.1)	26		−1.0 (−1.7– −0.3)
Mass Z-score at worst flare	0.392	0.433	0.295	89		−1.0 (−1.4–−0.2)	53		−0.7 (−1.3–0.1)	24		−1.0 (−1.4– −0.5)
Height Z-score at worst flare	0.339	0.602	0.359	90		−0.5 (−1.3–0.2)	53		−0.2 (−1.1–0.4)	24		−0.6 (−1.5–0.6)
BMI Z-score at worst flare	0.296	0.585	0.304	89		−0.9 (−1.6–0.0)	53		−0.7 (−1.2–0.4)	24		−1.0 (−1.3– −0.2)
PCDAI at diagnosis	0.387	0.418	0.282	93		35.0 (22.5–45.0)	57		25.0 (20.0–43.0)	26		35.0 (20.6–50.0)
PCDAI at worst flare	0.997	0.066	0.123	85		40.0 (30.0–52.5)	66		37.3 (25.0–51.3)	23		50.0 (36.3–55.0)
Systemic steroids	0.031 *	1		104	62.5%		66	42.4%		27	55.6%	
Azathioprine	0.159	0.798		104	83.7%		66	74.2%		27	77.8%	
Methotrexate	0.800	0.709		104	7.7%		66	9.1%		27	11.1%	
Ciclosporin	0.685	0.592		104	3.8%		66	1.5%		27	3.7%	
Biologics	0.089	0.680		104	55.8%		66	40.9%		27	55.6%	
Infliximab	0.123	0.536		103	53.4%		66	39.4%		27	55.6%	
Adalimumab	0.482	0.703		103	8.7%		25	9.1%		27	3.7%	
Months from diagnosis to biological therapy	0.394	0.435	0.249	56		17.6 (5.8–33.1)	25		11.0 (7.0–19.5)	15		23.4 (8.4–36.6)
Age at first biological therapy	0.850	0.471	0.748	56		13.8 (11.8–14.8)	66		13.7 (11.3–15.2)	15		13.5 (12.8–16.1)
IBD-related surgery	0.207	0.215		104	16.3%		61	12.1%		27	3.7%	
Times hospitalized for IBD flare	0.734	0.347	0.467	88		1.0 (1.0–2.0)	45		1.0 (1.0–2.0)	23		2.0 (1.0–2.5)
Hospitalizations for IBD flare per year	0.501	0.855	0.693	65		0.5 (0.2–0.9)	45		0.4 (0.2–0.8)	20		0.5 (0.3–0.8)
Days hospitalized for IBD flare per year	0.159	0.734	0.214	64		4.8 (2.0–7.6)	66		2.0 (1.0–6.6)	20		4.3 (1.8–6.5)

BMI—body mass index; CRP—C-reactive protein; IBD—inflammatory bowel disease; PCDAI—Pediatric Crohn’s Disease Activity Index.

**Table 4:** Characteristics of pediatric patients with inflammatory bowel disease dependent on *MST1* rs3197999 genotype. The Mann–Whitney U test, the Kruskal–Wallis test (K-W: comparison of three groups), and Fisher’s test were used for comparisons. Q1–Q3: quartiles 1–3. Statistically significant results are marked with an asterisk (*).

				CC			CT			TT		
Analyzed Variables	CC vs. Other *p*	TT vs. Other *p*	K-W *p*	n	%	Median (Q1–Q3)	n	%	Median (Q1–Q3)	n	%	Median (Q1–Q3)
Age at diagnosis [years]	0.629	0.271	0.333	191		11.9 (8.6–14.5)	130		12.5 (9.5–14.6)	44		11.7 (8.9–13.3)
Age at inclusion [years]	0.314	0.464	0.561	189		14.9 (12.7–16.8)	127		15.5 (12.6–16.9)	42		15.3 (12.7–17.4)
Duration of the disease [years]	0.474	0.102	0.090	184		2.2 (0.9–4.8)	125		2.3 (0.5–3.6)	42		3.3 (1.4–4.5)
Female	0.398	0.255		192	45.3%		131	42.7%		44	34.1%	
CRP at diagnosis [mg/L]	0.241	0.023 *	0.072	182		4.3 (0.7–21.8)	127		5.3 (1.3–17.9)	40		12.2 (3.0–32.9)
CRP at worst flare [mg/L]	0.683	0.305	0.589	161		4.8 (1.4–25.0)	116		6.5 (1.2–22.1)	35		9.2 (1.6–27.7)
Albumin at diagnosis [g/dL]	0.560	0.632	0.633	158		4.0 (3.4–4.4)	115		4.1 (3.7–4.4)	37		3.9 (3.7–4.2)
Albumin at worst flare [g/dL]	0.934	0.736	0.942	148		4.0 (3.6–4.3)	112		4.0 (3.5–4.4)	36		4.0 (3.6–4.3)
Mass Z-score at diagnosis	0.305	0.293	0.173	178		−0.7 (−1.3–0.0)	125		−0.5 (−1.1–0.2)	42		−0.8 (−1.4– 0.0)
Height Z-score at diagnosis	0.099	0.766	0.154	175		−0.3 (−1.1–0.4)	124		0.1 (−0.8–0.8)	42		−0.3 (−1.2–0.7)
BMI Z-score at diagnosis	0.570	0.623	0.631	175		−0.6 (−1.2–0.2)	124		−0.5 (−1.0–0.1)	42		−0.7 (−1.5–0.1)
Mass Z-score at worst flare	0.151	0.435	0.131	156		−0.8 (−1.3–0.0)	114		−0.6 (−1.0–0.5)	35		−0.8 (−1.3– −0.2)
Height Z-score at worst flare	0.016 *	0.983	0.035	157		−0.4 (−1.2–0.4)	114		−0.1 (−0.7–0.8)	35		0.0 (1.2–0.7)
BMI Z-score at worst flare	0.434	0.453	0.392	155		−0.7 (−1.3–0.1)	114		−0.7 (−1.2–0.4)	35		−0.8 (−1.2– −0.1)
Systemic steroids	0.329	0.867		192	66.1%		131	59.5%		44	65.9%	
Azathioprine	0.305	0.728		192	72.4%		130	65.4%		44	72.7%	
Methotrexate	0.671	0.501		192	5.7%		130	6.2%		44	9.1%	
Ciclosporin	0.300	1		192	8.3%		130	4.6%		44	6.8%	
Biologics	0.456	0.255		192	41.7%		131	34.4%		44	47.7%	
Infliximab	0.386	0.185		191	40.3%		128	31.3%		44	47.7%	
Adalimumab	0.685	0.754		191	7.9%		128	7.0%		44	4.5%	
Months from diagnosis to biological therapy	0.357	0.965	0.565	77		14.6 (6.3–29.9)	42		12.1 (7.9–24.0)	21		14.1 (4.7–29.1)
Age at first biological therapy	0.553	0.711	0.640	77		13.6 (10.9–15.3)	43		12.8 (10.5–15.1)	21		13.4 (10.8–15.8)
IBD-related surgery	0.447	1		192	9.4%		131	6.9%		44	6.6%	
Times hospitalized for IBD flare	0.691	0.707	0.791	162		2.0 (1.0–3.0)	123		1.0 (1.0–2.5)	35		2.0 (1.0–2.5)
Hospitalizations for IBD flare per year	0.463	0.878	0.755	113		0.6 (0.2–0.9)	82		0.6 (0.3–1.0)	29		0.6 (0.3–0.9)
Days hospitalized for IBD flare per year	0.935	0.548	0.784	112		4.6 (1.5–7.7)	82		3.5 (1.3–7.7)	29		4.9 (2.5–8.0)

BMI—body mass index; CRP—C-reactive protein; IBD—inflammatory bowel disease.

## Data Availability

The data presented in this study are available upon request from the corresponding author upon reasonable request.

## Supplementary Materials

The following supporting information can be downloaded at https://www.mdpi.com/article/10.3390/medicina60081243/s1: Figure S1: Allele discrimination; Table S1: List of abbreviations.

## References

[1] Zagórowicz E., Walkiewicz D., Kucha P., Perwieniec J., Maluchnik M., Wieszczy P., Reguła J. (2022). Nationwide Data on Epidemiology of Inflammatory Bowel Disease in Poland between 2009 and 2020. Pol. Arch. Intern. Med..

[2] Ng S.C., Shi H.Y., Hamidi N., Underwood F.E., Tang W., Benchimol E.I., Panaccione R., Ghosh S., Wu J.C.Y., Chan F.K.L. (2018). Worldwide Incidence and Prevalence of Inflammatory Bowel Disease in the 21st Century: A Systematic Review of Population-Based Studies. Lancet.

[3] Monstad I.L., Solberg I.C., Cvancarova M., Hovde O., Henriksen M., Huppertz-Hauss G., Gunther E., Moum B.A., Stray N., Vatn M. (2021). Outcome of Ulcerative Colitis 20 Years after Diagnosis in a Prospective Population-Based Inception Cohort from South-Eastern Norway, the IBSEN Study. J. Crohns Colitis.

[4] Buie M.J., Quan J., Windsor J.W., Coward S., Hansen T.M., King J.A., Kotze P.G., Gearry R.B., Ng S.C., Mak J.W.Y. (2023). Global Hospitalization Trends for Crohn’s Disease and Ulcerative Colitis in the 21st Century: A Systematic Review with Temporal Analyses. Clin. Gastroenterol. Hepatol..

[5] Zhao M., Gönczi L., Lakatos P.L., Burisch J. (2021). The Burden of Inflammatory Bowel Disease in Europe in 2020. J. Crohns Colitis.

[6] Chanchlani N., Lin S., Bewshea C., Hamilton B., Thomas A., Smith R., Roberts C., Bishara M., Nice R., Lees C.W. (2024). Mechanisms and Management of Loss of Response to Anti-TNF Therapy for Patients with Crohn’s Disease: 3-Year Data from the Prospective, Multicentre PANTS Cohort Study. Lancet Gastroenterol. Hepatol..

[7] Yanai H., Levine A., Hirsch A., Boneh R.S., Kopylov U., Eran H.B., Cohen N.A., Ron Y., Goren I., Leibovitzh H. (2022). The Crohn’s Disease Exclusion Diet for Induction and Maintenance of Remission in Adults with Mild-to-Moderate Crohn’s Disease (CDED-AD): An Open-Label, Pilot, Randomised Trial. Lancet Gastroenterol. Hepatol..

[8] Svolos V., Hansen R., Nichols B., Quince C., Ijaz U.Z., Papadopoulou R.T., Edwards C.A., Watson D., Alghamdi A., Brejnrod A. (2019). Treatment of Active Crohn’s Disease with an Ordinary Food-Based Diet That Replicates Exclusive Enteral Nutrition. Gastroenterology.

[9] Kedia S., Virmani S., Vuyyuru S.K., Kumar P., Kante B., Sahu P., Kaushal K., Farooqui M., Singh M., Verma M. (2022). Faecal Microbiota Transplantation with Anti-Inflammatory Diet (FMT-AID) Followed by Anti-Inflammatory Diet Alone Is Effective in Inducing and Maintaining Remission over 1 Year in Mild to Moderate Ulcerative Colitis: A Randomised Controlled Trial. Gut.

[10] Papadimitriou K. (2022). The Influence of Aerobic Type Exercise on Active Crohn’s Disease Patients: The Incidence of an Elite Athlete. Healthcare.

[11] Prins F.M., Hidding I.J., Klaassen M.A.Y., Collij V., Schultheiss J.P.D., Venema W.T.C.U., Bangma A., Aardema J.B., Jansen B.H., Mares W.G.N. (2024). Limited Predictive Value of the Gut Microbiome and Metabolome for Response to Biological Therapy in Inflammatory Bowel Disease. medRxiv.

[12] Shubhakar A., Jansen B.C., Adams A.T., Reiding K.R., Ventham N.T., Kalla R., Bergemalm D., Urbanowicz P.A., Gardner R.A., Consortium I.-B. (2023). Serum N-Glycomic Biomarkers Predict Treatment Escalation in Inflammatory Bowel Disease. J. Crohns Colitis.

[13] de Lange K.M., Moutsianas L., Lee J.C., Lamb C.A., Luo Y., Kennedy N.A., Jostins L., Rice D.L., Gutierrez-Achury J., Ji S.-G. (2017). Genome-Wide Association Study Implicates Immune Activation of Multiple Integrin Genes in Inflammatory Bowel Disease. Nat. Genet..

[14] Goyette P., Lefebvre C., Ng A., Brant S.R., Cho J.H., Duerr R.H., Silverberg M.S., Taylor K.D., Latiano A., Aumais G. (2008). Gene-Centric Association Mapping of Chromosome 3p Implicates MST1 in IBD Pathogenesis. Mucosal Immunol..

[15] Melum E., Franke A., Schramm C., Weismüller T.J., Gotthardt D.N., Offner F.A., Juran B.D., Laerdahl J.K., Labi V., Björnsson E. (2011). Genome-Wide Association Analysis in Primary Sclerosing Cholangitis Identifies Two Non-HLA Susceptibility Loci. Nat. Genet..

[16] Karlsen T.H., Franke A., Melum E., Kaser A., Hov J.R., Balschun T., Lie B.A., Bergquist A., Schramm C., Weismüller T.J. (2010). Genome-Wide Association Analysis in Primary Sclerosing Cholangitis. Gastroenterology.

[17] Häuser F., Deyle C., Berard D., Neukirch C., Glowacki C., Bickmann J.K., Wenzel J.J., Lackner K.J., Rossmann H. (2012). Macrophage-Stimulating Protein Polymorphism Rs3197999 Is Associated with a Gain of Function: Implications for Inflammatory Bowel Disease. Genes. Immun..

[18] Li T., Wen Y., Lu Q., Hua S., Hou Y., Du X., Zheng Y., Sun S. (2023). MST1/2 in Inflammation and Immunity. Cell Adhes. Migr..

[19] Shao Y., Wang Y., Sun L., Zhou S., Xu J., Xing D. (2023). MST1: A Future Novel Target for Cardiac Diseases. Int. J. Biol. Macromol..

[20] Wang Y., Jia A., Cao Y., Hu X., Wang Y., Yang Q., Bi Y., Liu G. (2020). Hippo Kinases MST1/2 Regulate Immune Cell Functions in Cancer, Infection, and Autoimmune Diseases. Crit. Rev. Eukaryot. Gene Expr..

[21] Zinatizadeh M.R., Miri S.R., Zarandi P.K., Chalbatani G.M., Rapôso C., Mirzaei H.R., Akbari M.E., Mahmoodzadeh H. (2021). The Hippo Tumor Suppressor Pathway (YAP/TAZ/TEAD/MST/LATS) and EGFR-RAS-RAF-MEK in Cancer Metastasis. Genes Dis..

[22] Zhou D., Medoff B.D., Chen L., Li L., Zhang X., Praskova M., Liu M., Landry A., Blumberg R.S., Boussiotis V.A. (2008). The Nore1B/Mst1 Complex Restrains Antigen Receptor-Induced Proliferation of Naïve T Cells. Proc. Natl. Acad. Sci. USA.

[23] Ueda Y., Katagiri K., Tomiyama T., Yasuda K., Habiro K., Katakai T., Ikehara S., Matsumoto M., Kinashi T. (2012). Mst1 Regulates Integrin-Dependent Thymocyte Trafficking and Antigen Recognition in the Thymus. Nat. Commun..

[24] Li J., Du X., Shi H., Deng K., Chi H., Tao W. (2015). Mammalian Sterile 20-like Kinase 1 (Mst1) Enhances the Stability of Forkhead Box P3 (Foxp3) and the Function of Regulatory T Cells by Modulating Foxp3 Acetylation. J. Biol. Chem..

[25] Cheng J., Jing Y., Kang D., Yang L., Li J., Yu Z., Peng Z., Li X., Wei Y., Gong Q. (2018). The Role of Mst1 in Lymphocyte Homeostasis and Function. Front. Immunol..

[26] Chen J., Xu F., Ruan X., Sun J., Zhang Y., Zhang H., Zhao J., Zheng J., Larsson S.C., Wang X. (2023). Therapeutic Targets for Inflammatory Bowel Disease: Proteome-Wide Mendelian Randomization and Colocalization Analyses. eBioMedicine.

[27] Levine A., Koletzko S., Turner D., Escher J.C., Cucchiara S., de Ridder L., Kolho K.-L., Veres G., Russell R.K., Paerregaard A. (2014). ESPGHAN Revised Porto Criteria for the Diagnosis of Inflammatory Bowel Disease in Children and Adolescents. J. Pediatr. Gastroenterol. Nutr..

[28] Sawicka-Gutaj N., Gruszczyński D., Guzik P., Mostowska A., Walkowiak J. (2022). Publication Ethics of Human Studies in the Light of the Declaration of Helsinki—A Mini-Review. J. Med. Sci..

[29] Turner D., Otley A.R., Mack D., Hyams J., De Bruijne J., Uusoue K., Walters T.D., Zachos M., Mamula P., Beaton D.E. (2007). Development, Validation, and Evaluation of a Pediatric Ulcerative Colitis Activity Index: A Prospective Multicenter Study. Gastroenterology.

[30] Hyams J.S., Ferry G.D., Mandel F.S., Gryboski J.D., Kibort P.M., Kirschner B.S., Griffiths A.M., Katz A.J., Grand R.J., Boyle J.T. (1991). Development and Validation of a Pediatric Crohn’s Disease Activity Index. J. Pediatr. Gastroenterol. Nutr..

[31] Shamir R., Shamir R., Turck D., Phillip M. (2013). Nutrition and Growth in Inflammatory Bowel Disease. World Review of Nutrition and Dietetics.

[32] Cleynen I., Boucher G., Jostins L., Schumm L.P., Zeissig S., Ahmad T., Andersen V., Andrews J.M., Annese V., Brand S. (2016). Inherited Determinants of Crohn’s Disease and Ulcerative Colitis Phenotypes: A Genetic Association Study. Lancet.

[33] Palmieri O., Bossa F., Valvano M.R., Corritore G., Latiano T., Martino G., D’Incà R., Cucchiara S., Pastore M., D’Altilia M. (2017). Crohn’s Disease Localization Displays Different Predisposing Genetic Variants. PLoS ONE.

[34] Latiano A., Palmieri O., Corritore G., Valvano M.R., Bossa F., Cucchiara S., Castro M., Riegler G., De Venuto D., D’Incà R. (2010). Variants at the 3p21 Locus Influence Susceptibility and Phenotype Both in Adults and Early-Onset Patients with Inflammatory Bowel Disease. Inflamm. Bowel Dis..

[35] Kaur M., Panikkath D., Yan X., Liu Z., Berel D., Li D., Vasiliauskas E.A., Ippoliti A., Dubinsky M., Shih D.Q. (2016). Perianal Crohn’s Disease Is Associated with Distal Colonic Disease, Stricturing Disease Behavior, IBD-Associated Serologies and Genetic Variation in the JAK-STAT Pathway. Inflamm. Bowel Dis..

[36] Wu W.K.K., Sun R., Zuo T., Tian Y., Zeng Z., Ho J., Wu J.C.Y., Chan F.K.L., Chan M.T.V., Yu J. (2018). A Novel Susceptibility Locus in MST1 and Gene-Gene Interaction Network for Crohn’s Disease in the Chinese Population. J. Cell Mol. Med..

[37] Niriella M.A., Liyanage I.K., Kodisinghe S.K., Silva A.P.D., Rajapakshe N., Nanayakkara S.D., Luke D., Silva T., Nawarathne M., Peiris R.K. (2018). Genetic Associations of Inflammatory Bowel Disease in a South Asian Population. World J. Clin. Cases.

[38] Skieceviciene J., Kiudelis G., Ellinghaus E., Balschun T., Jonaitis L.V., Zvirbliene A., Denapiene G., Leja M., Pranculiene G., Kalibatas V. (2013). Replication Study of Ulcerative Colitis Risk Loci in a Lithuanian-Latvian Case-Control Sample. Inflamm. Bowel Dis..

[39] Di Narzo A.F., Telesco S.E., Brodmerkel C., Argmann C., Peters L.A., Li K., Kidd B., Dudley J., Cho J., Schadt E.E. (2017). High-Throughput Characterization of Blood Serum Proteomics of IBD Patients with Respect to Aging and Genetic Factors. PLoS Genet..

[40] Gorlatova N., Chao K., Pal L.R., Araj R.H., Galkin A., Turko I., Moult J., Herzberg O. (2011). Protein Characterization of a Candidate Mechanism SNP for Crohn’s Disease: The Macrophage Stimulating Protein R689C Substitution. PLoS ONE.

[41] Krawczyk M., Höblinger A., Mihalache F., Grünhage F., Acalovschi M., Lammert F., Zimmer V. (2013). Macrophage Stimulating Protein Variation Enhances the Risk of Sporadic Extrahepatic Cholangiocarcinoma. Dig. Liver Dis..

[42] Maxwell T.J., Franks P.W., Kahn S.E., Knowler W.C., Mather K.J., Florez J.C., Jablonski K.A., and for the Diabetes Prevention Program Research Group (2022). Quantitative Trait Loci, G×E and G×G for Glycemic Traits: Response to Metformin and Placebo in the Diabetes Prevention Program (DPP). J. Hum. Genet..

[43] Laukens D., Georges M., Libioulle C., Sandor C., Mni M., Vander Cruyssen B., Peeters H., Elewaut D., De Vos M. (2010). Evidence for Significant Overlap between Common Risk Variants for Crohn’s Disease and Ankylosing Spondylitis. PLoS ONE.

[44] Zou M., Liang Q., Zhang W., Zhu Y., Xu Y. (2023). Endoplasmic Reticulum Stress Related Genome-Wide Mendelian Randomization Identifies Therapeutic Genes for Ulcerative Colitis and Crohn’s Disease. Front. Genet..

[45] Costa Pereira C., Durães C., Coelho R., Grácio D., Silva M., Peixoto A., Lago P., Pereira M., Catarino T., Pinho S. (2017). Association between Polymorphisms in Antioxidant Genes and Inflammatory Bowel Disease. PLoS ONE.

[46] Chu F.-F., Esworthy R.S., Shen B., Doroshow J.H. (2020). Role of the Microbiota in Ileitis of a Mouse Model of Inflammatory Bowel Disease-Glutathione Peroxide Isoenzymes 1 and 2-Double Knockout Mice on a C57BL Background. Microbiologyopen.

[47] Mrowicka M., Mrowicki J., Mik M., Dziki Ł., Dziki A., Majsterek I. (2020). Assessment of DNA Damage Profile and Oxidative /Antioxidative Biomarker Level in Patients with Inflammatory Bowel Disease. Pol. Przegl Chir..

[48] Eriksson A., Flach C.-F., Lindgren A., Kvifors E., Lange S. (2008). Five Mucosal Transcripts of Interest in Ulcerative Colitis Identified by Quantitative Real-Time PCR: A Prospective Study. BMC Gastroenterol..

