# Supplementary material for: Macrophage-Stimulating 1 Polymorphism rs3197999 in Pediatric Patients with Inflammatory Bowel Disease
Source: Medicina (Kaunas). 2024 Jul 31;60(8):1243. doi: 10.3390/medicina60081243 (PMC11356727; doi:10.3390/medicina60081243)
Supplement: Supplementary file 1 [file medicina-60-01243-s001.zip › Supplementary Table S1.pdf]

Supplementary Table S1. List of abbreviations.

Abbreviation	Meaning
APEH	acylaminoacyl-peptide hydrolase
BMI	body mass index
CARD9	caspase recruitment domain-containing protein 9
CC	cytosine/cytosine
CD	Crohn's disease
CRP	C-reactive protein
CT	cytosine/thymine
DNA	deoxyribonucleic acid
EDTA	ethylenediaminetetraacetic acid
FoxP3	forkhead box P3
GPX1	glutathione peroxidase 1
IBD	Inflammatory bowel disease
ICAM-1	intercellular adhesion molecule 1
IL10	interleukin-10
IL23R	interleukin-23 receptor
K-W	Kruskal-Wallis test
LFA-1	lymphocyte function-associated antigen 1
MST1	macrophage-stimulating 1
NOD2	nucleotide-binding oligomerization domain containing 2
OR	odds ratio
PCDAI	Pediatric Crohn's Disease Activity Index
PCR	polymerase chain reaction
PUCAI	Pediatric Ulcerative Colitis Activity Index
Q1–Q3	quartiles 1-3
SNP	single nucleotide polymorphism
TNF-alpha	tumor necrosis factor alpha
TT	thymine/thymine
UC	ulcerative colitis
