# Supplementary figures and images for: Macrophage-Stimulating 1 Polymorphism rs3197999 in Pediatric Patients with Inflammatory Bowel Disease
Source: Medicina (Kaunas). 2024 Jul 31;60(8):1243. doi: 10.3390/medicina60081243 (PMC11356727; doi:10.3390/medicina60081243)

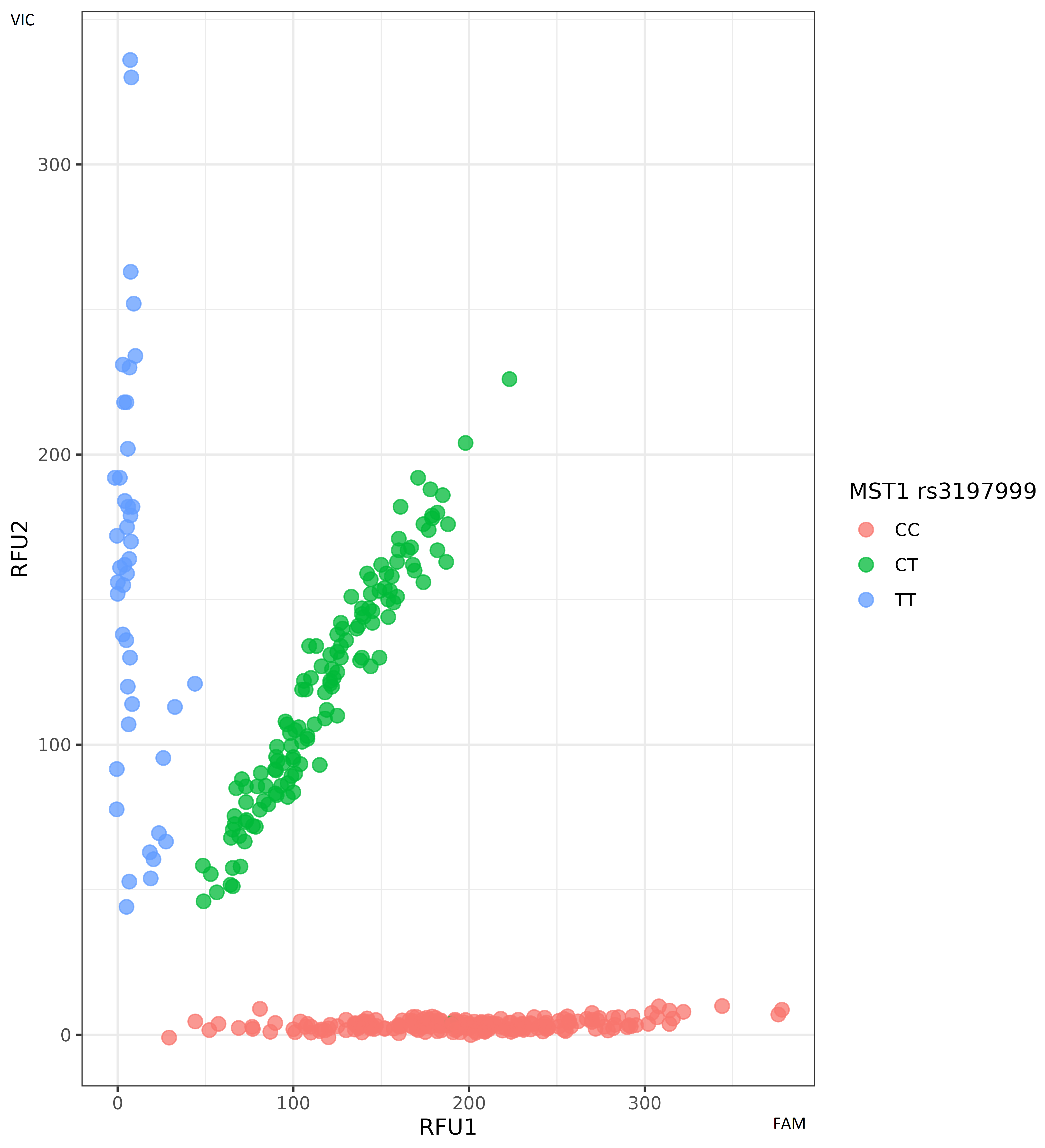

Supplement: Supplementary file 1 [file medicina-60-01243-s001.zip › Supplementary Figure S1.png]
